# India Declines Patent Extension Application of Bedaquiline: A Remarkable Step Towards Tuberculosis Elimination

**DOI:** 10.7759/cureus.48146

**Published:** 2023-11-02

**Authors:** Sankalp Yadav

**Affiliations:** 1 Medicine, Shri Madan Lal Khurana Chest Clinic, New Delhi, IND

**Keywords:** johnson & johnson, mycobacterium tuberculosis (mtb), patent, extensively drug resistant (xdr), mdr-tb (multi drug resistant tuberculosis), tuberculosis, bedaquiline

## Abstract

In a pivotal decision, an application to extend the patent on bedaquiline submitted by the pharmaceutical company Johnson & Johnson was denied by the Indian Patent Office, an essential drug for the treatment of tuberculosis. This development marks the conclusion of eight years of drug exclusivity, potentially opening the door for generic and more affordable versions. Bedaquiline has been a game-changer in treating multidrug-resistant tuberculosis and extensively drug-resistant tuberculosis and has shown remarkable clinical efficacy. The refusal to extend the patent aligns with India's goal to eliminate TB by 2025 and may significantly contribute to making bedaquiline more accessible to those in need. This decision carries broader implications, establishing a precedent for improved availability of reasonably priced drugs, diagnostics, and vaccines for illnesses with widespread outbreaks in nations with limited resources. While patent protection is vital for stimulating innovation, mechanisms to ensure global accessibility to essential medications remain imperative. Further reduction of costs and growth of facilities for manufacturing to low- and middle-income countries are essential steps in this journey. This paper highlights the potential impact of the Indian Patent Office's decision on TB management, access to treatment, and global health initiatives.

## Editorial

Developing nations are experiencing a sharp rise in the incidence of drug-resistant tuberculosis (DR-TB) cases [1]. When a *Mycobacterium tuberculosis* strain is resistant to no less than two of the four main TB treatments, isoniazid and rifampicin, it is referred to be multidrug-resistant tuberculosis (MDR-TB) with pyrazinamide and ethambutol being the other two medications [1].

Managing these patients is complex and requires a 24-to 27-month course of treatment. Until 2016, the guidelines for managing this strain of TB primarily relied on second-line TB medications, which were relatively expensive, less effective, and linked to more side effects [1]. To address the high death rate and attain timely sputum culture conversion in instances with MDR-TB, it was necessary to provide new medications.

DR-TB has become a serious public health issue since 1990 [1]. According to WHO estimates from 2013, the prevalence of primary MDR-TB in India is approximately 3.5%. However, this prevalence rises to 20.5% among cases that have been previously treated [1]. This indicates that MDR-TB primarily arises due to human actions, particularly improper or inadequately administered treatment, and is the result of spontaneous mutations in the bacilli's genes [1]. Furthermore, TB infection demographics vary considerably, with a more pronounced burden on developing countries [1].

One of bedaquiline's notable features is its novel mechanism of action, reducing its susceptibility to developing cross-resistance with other antitubercular drugs commonly used for MDR-TB. Moreover, bedaquiline possesses a protracted half-life of ultimate elimination, practically 5.5 months because of a combination of strong tissue penetration, extended tissue half-lives in plasma, and *Mycobacterium tuberculosis*-affected organs [1].

On December 28, 2012, the Food and Drug Administration (FDA) granted accelerated approval to SIRTURO™ (bedaquiline) tablets for inclusion in second-line antitubercular chemotherapy for MDR-TB in adults [2]. This expedited approval signified the FDA's recognition that the therapeutic benefits of bedaquiline should be made available to the impacted population. As a result, bedaquiline (formerly known as TMC207) became the first newly approved anti-TB drug since rifapentine in 1998 [2]. Notably, bedaquiline is the first anti-TB medication with a novel mechanism of action to gain approval in over 40 years, with rifampicin having been approved in 1974 [1,2]. Bedaquiline represents a pioneering introduction tailored specifically for the management of MDR-TB in combination with other medications [2]. It is a member of the diarylquinoline class of anti-TB pharmaceuticals, which bonds to the protein subunit c to inhibit the function of the mycobacterial ATP synthase enzyme [2]. The medication is effective against mycobacteria that are actively replicating as well as those that are not. A study showed that even at low doses, it can inhibit dormant cells in latent TB infection [1]. As a result, it displays potent bactericidal and sterilizing properties and exhibits high plasma binding. Bedaquiline undergoes liver metabolism and is primarily excreted through feces [1,2].

Bedaquiline demonstrated activity against drug-sensitive, MDR, pre-extensively drug-resistant (pre-XDR), and extensively drug-resistant (XDR) strains of *Mycobacterium tuberculosis* in vitro [1,2]. In India's TB elimination program, bedaquiline was first introduced on March 21, 2016 [1]. The drug has shown promising outcomes, including early culture conversion, as indicated by earlier reports [1]. Given the continuously increasing numbers of MDR-TB cases, there was a pressing demand to implement bedaquiline widely throughout the entire country. Hence, it became an essential part of all DR-TB regimens.

During the subsequent seven years of clinical trials, the evaluation revealed that bedaquiline was a transformative development, significantly enhancing treatment outcomes for individuals with MDR-TB and XDR-TB [3]. Pharmaceutical companies, including Johnson & Johnson, which held the patent rights to the drug, had full control over determining its high price. In October 2019, Médecins Sans Frontières (MSF), TB advocates, and civil society groups started a worldwide campaign, staging protests outside Johnson & Johnson offices in the United States, Brazil, South Africa, Ukraine, Belgium, and Spain. They demanded that the cost of bedaquiline for patients with MDR-TB be lowered to not exceed one dollar per day, enabling a rapid expansion of treatment and the ability to render patients non-infectious, effectively halting the spread of the disease within the population [3].

In the wake of these demands, the Indian Patent Office, Mumbai has declined an application from Johnson & Johnson on March 23, 2023, to prolong the patent for bedaquiline past July 2023. This decision effectively marks the conclusion of their eight-year exclusive rights to the TB drug. It also paves the way for pharmaceutical companies to manufacture more affordable, generic editions of bedaquiline, which is expected to assist India in achieving its objective of eradicating TB by 2025 [4]. The Indian patent office's choice provides an opportunity for other companies to manufacture more affordable and readily available iterations of bedaquiline [3].

The pharmaceutical company Johnson & Johnson argued that patents were the only way to cover the enormous expense of creating a new medication. But their patent remained valid for 20 years which was a substantial amount to recover the expenses. Besides, an extension of the patent for the fumarate salt when lives are on the line was unacceptable. Additionally, the patent of bedaquiline is also opposed in Belarus, Brazil, India, Kyrgyzstan, Moldova, Republic of Thailand, Ukraine, and Vietnam.

In 2018, the price of a six-month course of bedaquiline decreased from $900 to $400. Nevertheless, the drug has been administered to about 10,000 individuals in India. Medical professionals think that with further cost reductions, a larger population could benefit from access to the drug [4]. According to some health professionals, this move could potentially reduce treatment expenses by up to 80%, lowering the cost per patient from $46 (€42.6) to $8 (€7.4) per month [3].

The momentous ruling from the Indian patent office not only paves the path for more affordable bedaquiline variants to be produced but also for them to become more accessible, particularly in regions with limited resources and high rates of MDR-TB. Furthermore, it sets a significant precedent for broadening access to reasonably priced drugs, diagnostic tools, and vaccines for other epidemic diseases that disproportionately impact resource-scarce nations. The India TB Report 2023 reported that there was a remarkable rise in the cases of DR-TB in the year 2022 as compared to 2021 [5]. A similar incident happened in April 2021, when a Brazilian judge issued a preliminary decision that suspended drug patent extensions, potentially reducing the costs of essential medications for treating coronavirus disease 2019 patients, albeit at the price of drug manufacturers [3]. Nevertheless, three Indian firms i.e., Lupin, Natco, and Macleods lined up to make the generic versions of bedaquiline. Also, there are a number of bedaquiline analogues/derivatives the closest being analogue 17, which could be of interest to these companies.

In summary, while upholding patent rights remains crucial to incentivize the creation of novel drugs, vaccines, and diagnostics, there is an urgent requirement for mechanisms that guarantee sufficient global access. This entails the extension of production capabilities to low- and middle-income countries, creating job opportunities and fostering professional growth, along with donation initiatives sponsored by affluent nations and price reductions through bulk purchasing arrangements. Moreover, the uninterrupted supply of highly effective drugs like bedaquiline at cheaper rates would have a tremendous impact on the ultimate aim of TB elimination from India. 

## References

[1] Yadav S, Rawal G, Baxi M (2016). Bedaquiline: a novel antitubercular agent for the treatment of multidrug-resistant tuberculosis. J Clin Diagn Res.

[2] Deoghare S (2013). Bedaquiline: a new drug approved for treatment of multidrug-resistant tuberculosis. Indian J Pharmacol.

[3] Petersen E, Hui DS, Nachega JB (2023). End of the bedaquiline patent - a crucial development for moving forward affordable drugs, diagnostics, and vaccines for infectious diseases in low- and middle-income countries. Int J Infect Dis.

[4] Thiagarajan K (2023). India rejects application to extend patent on TB drug bedaquiline. BMJ.

[5] (2023). India TB report 2023. https://tbcindia.gov.in/showfile.php.

